# New Treatment Options for Late Na Current, Arrhythmias, and Diastolic Dysfunction

**DOI:** 10.1007/s11897-012-0099-3

**Published:** 2012-07-06

**Authors:** Lars S. Maier

**Affiliations:** Abteilung Kardiologie und Pneumologie/Herzzentrum, Deutsches Zentrum für Herzkreislaufforschung, Georg-August-Universität Göttingen, Robert-Koch-Strasse 40, 37075 Göttingen, Germany

**Keywords:** Late Na current, Intracellular Na overload, Late Na current inhibition, Ranolazine, Intracellular Ca overload, Diastolic dysfunction, Heart failure, Heart failure with preserved ejection fraction, Action potential duration, Arrhythmias, Atrial fibrillation, Ventricular arrhythmias, Treatment, Therapy

## Abstract

The late Na current is of pathophysiological importance for the heart. Ranolazine is an innovative anti-ischemic and antianginal agent that inhibits the late Na current, thereby reducing the Na-dependent Ca-overload, which improves diastolic tone and oxygen handling during myocardial ischemia. In addition, ranolazine seems to exert beneficial effects on diastolic cardiac function. Moreover, there are experimental and clinical data about its antiarrhythmic properties. A beneficial atrial selectivity of ranolazine has been suggested that may be helpful for the treatment of atrial fibrillation. The purpose of this review article is to discuss possible future clinical indications based on novel experimental and preclinical results and the significance of the available data.

## Introduction: The Late Na Current

Under physiological conditions, sarcolemmal Na channels open transiently and are quickly inactivated, thereby producing the peak Na current (I_Na_) and, thus, the upstroke of the action potential. In addition, a late component of I_Na_ was described due to Na channels that remain active, inactivate with much slower kinetics, or reopen. The amplitude of this current is small with about 1 % of the amplitude of peak I_Na_, but it may persist for hundreds of milliseconds. Because late I_Na_ can be elevated up to about 5 times under pathological conditions [1], one can imagine that the integral of this persistent current may exceed the one of peak I_Na_ leading to Na accumulation in the myocyte. It is known that intracellular Na concentration frequency dependently can increase by several mM [2]. In human heart failure, intracellular Na concentration can be up to 6–8 mM higher as compared to nonfailing myocardium [3].

Different Na channel isoforms are known (eg, Na_v_1.1–Na_v_1.8), with the pore-forming subunit Na_v_1.5 being the cardiac specific isoform. The contribution of Na channel isoforms for late I_Na_ and its modulation by different pathological conditions are not fully understood. Most of the studies identified Na_v_1.5 as the late I_Na_ producing channel [4]. Auxiliary ß subunits also exist [5]. There is one report that shows that neuronal isoforms Na_v_1.1 and Na_v_1.6 increase proportionally with increasing late I_Na_ in pressure-overloaded rat hearts [6]. Also, mutations in the Na channel gene SCN5A encoding for Na_v_1.5 that are associated with the long QT syndrome 3 produce slowed inactivation thereby increasing late I_Na_ [7].

Of broader clinical relevance are acquired disease states such as ischemia, myocardial infarction, and heart failure that are known to be associated with an elevated late I_Na_ [8–10]. We have shown that late I_Na_ is increased in myocytes from patients with atrial fibrillation (AF) [11••] and in isolated myocardium from patients with heart failure and diastolic dysfunction [12].

As mentioned above, an elevated late I_Na_ is considered to be a potent contributor of Na overload [12, 13]. Although Na overload itself cannot activate myofilaments directly, increasing Na levels can lead to Ca overload through the Na/Ca-exchanger (NCX). Normally, the NCX exchanges one Ca ion for three Na ions per cycle. However, the NCX can work in two different directions. In its forward mode, it eliminates Ca out of the cell to accomplish diastolic relaxation (in addition to sarcoplasmic reticulum Ca reuptake mainly through the sarcoplasmic reticulum Ca ATPase). In its reverse mode (usually during the action potential plateau), it transports Ca into the cell in exchange to transsarcolemmal elimination of Na. The activity and direction of the transport depends on the membrane potential and, thus, on the action potential as well as the intracellular Na and Ca concentration (ie, the electrochemical gradient). Na accumulation and prolonged action potential duration occur during myocardial hypoxia and promote reverse mode of the NCX [14]. The consequence is an impaired overall capacity of the cell to eliminate Ca from the cytosol leading to intracellular Ca overload. This leads to or aggravates diastolic dysfunction due to increased myofilaments activation [15]. Thus, diastolic activation of contractile proteins causing increased wall tension prolonging extravascular compression of intramural vessels reduces oxygen supply [16]. Additionally, elevated diastolic tone increases energy consumption and aggravates disturbed energy balance like a vicious cycle [17].

## Inhibition of Late I_Na_

Ranolazine is an inhibitor of late I_Na_ and is available for clinical purposes since 2006 as an anti-ischemic agent (Ranexa^®^ [Gilead Sciences, Inc., Foster City, CA]) [18, 19]. In cardiac myocytes from dogs and guinea pigs, ranolazine was shown to cause a concentration-, voltage-, and frequency-dependent inhibition of late I_Na_ [20]. Inhibitory effects of ranolazine on late I_Na_ were demonstrated in multicellular myocardium and in isolated cardiac myocytes [12, 21, 22]. Although ranolazine also has some peak I_Na_ inhibiting effects, it has an up to 38-fold higher potency for late I_Na_ as compared to peak I_Na_ with a half maximal inhibitory concentration (IC_50_) of 6.5 versus 244 μM [21] in ventricular myocytes with a therapeutic range of 2–8 μM. Regarding the binding site of ranolazine on the cardiac Na channel, two independent studies described mutation of a single amino acid residue, F1760A and F1759K, in the putative local anesthetic binding site of the cardiac Na channel Na_v_1.5 [7], and the skeletal muscle channel Na_v_1.4 [23], showing that this mutation reduced the late I_Na_ inhibiting effects of ranolazine. However, it remains unclear how ranolazine produces its selective effect on late I_Na_.

Interestingly, in two very recent studies, additional modes of action of ranolazine were suggested. Beyder et al. [24•] could show a mechanosensitivity of Na_v_1.5 in isolated cardiac myocytes and that ranolazine was able to reduce this novel activation mechanism. However, this effect seemed to be independent of the suspected binding site F1760. Therefore, the exact molecular mode of action remains unexplained. Of not, a stretch-dependent effect on intracellular Na handling was previously proposed by us mainly through reverse mode NCX [25]. In addition, Lovelock et al. [26] could describe an effect of ranolazine on myofilament Ca sensitivity, thereby improving diastolic function.

## Late I_Na_ Inhibition in Heart Failure: Effects on Cytosolic Na and Ca Handling

Diastolic heart failure is characterized by signs and symptoms of heart failure. These patients show a decreased compliance and relaxation of the ventricles and present with preserved ejection fraction (HFpEF). Unfortunately, there are no evidence-based agents for treating HFpEF. Because late I_Na_ is elevated in human heart failure [10], there is ongoing effort to investigate possible effects of ranolazine in conditions of heart failure.

Almost 20 years ago, there was an elegant in vivo study showing an improved diastolic function in noninfarcted ischemic hearts in a small number of patients before and in the presence of intravenous application of ranolazine [27]. Moreover, acute infusion of ranolazine in patients with long QT syndrome 3 with increased late I_Na_ caused a significant improvement of diastolic relaxation in parallel to an abbreviation of the QT time [28].

In addition, a recent echocardiographic study investigated the effects of ranolazine in patients with stable angina with preserved ejection fraction [29••]. After 2 months deceleration time E, isovolumic contraction time, and isovolumic relaxation time decreased, whereas ejection time increased. Global left ventricular function also improved, as indicated by a decrease in the myocardial performance index.

Interestingly, a subgroup analysis of the Metabolic Efficiency with Ranolazine for Less Ischemia in Non-ST Elevation Acute Coronary Syndromes (MERLIN TIMI-36) study revealed that patients with an acute coronary syndrome and elevated B-type natriuretic peptide levels (> 80 pg/mL), and thus increased wall stress, were at significantly higher risk of the primary trial end point of cardiovascular death and myocardial infarction at 1 year [30].

In contrast to the small number of clinical trials, there are a couple of experimental studies using ranolazine in vivo and in vitro in different heart failure models. First, in dogs with heart failure, acute infusion of ranolazine significantly reduced left ventricular end diastolic pressure, and increased ejection fraction as well as stroke volume [31]. Most importantly, these effects were observed in the absence of significant changes in heart rate or blood pressure. In another study, ranolazine was investigated as a chronic treatment option in a heart failure model again in dogs [32]. This study additionally examined the effects of ranolazine treatment in combination with the ß-blocker metoprolol or the angiotensin-converting enzyme (ACE) inhibitor enalapril. Ranolazine was capable to significantly prevent progressive left ventricular dysfunction as well as global and cellular myocardial remodeling.

**Fig. 1:**
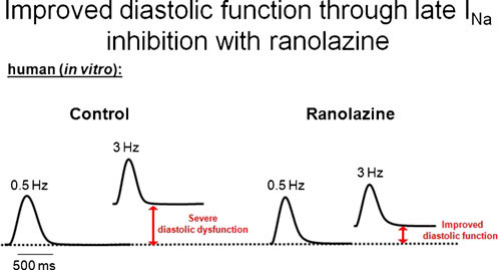
Original traces representing diastolic dysfunction in heart failure and improvement upon ranolazine (*Adapted from* Sossalla et al. [12].)

**Fig. 2:**
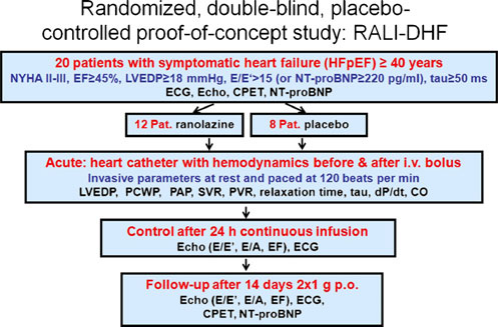
Schematic diagram showing the design of the RALI-DHF study. *RALI-DHF* Ranolazine in Diastolic Heart Failure; *NYHA* New York Heart Association class; *EF* ejection fraction; *LVEDP* left ventricular end diastolic pressure; *NT-proBNP* N-terminal pro–B-type natriuretic peptide; *ECG* electrocardiogram; *Echo* echocardiogram; *CPET* cardiopulmonary exercise testing; Pat patient; *i.v.* intravenous; *PCWP* pulmonary capillary wedge pressure; *PAP* pulmonary artery pressure; *SVR* systemic vascular resistance; *PVR* pulmonary vascular resistance; dP/dt; rate of pressure change in the ventricle

Our group investigated isolated trabeculae from human end-stage failing hearts that exhibited frequency-dependent diastolic dysfunction in vitro [12]. Addition of ranolazine did not cause negative inotropy but significantly ameliorated disturbed intracellular ion handling and reduced the increase in diastolic tension (ie, improved diastolic dysfunction [Fig. 1]). Interestingly, those trabeculae with severe diastolic dysfunction benefit from late I_Na_ inhibition even more. This hypothesis could be confirmed in experiments in isolated rabbit myocytes that were exposed to anemone toxin (ATX-II) to mimic increased late I_Na_ in heart failure. Using fluorescent dyes, the increase in cytosolic Na was paralleled by elevated intracellular diastolic Ca levels. Treatment with ranolazine significantly attenuated the effects of ATX-II leading to markedly reduced diastolic Ca levels in addition to lowering Na levels. Interestingly, ranolazine secondarily accelerated the NCX forward mode activity measured by analysis of Ca transients decay during caffeine applications [12]. This increase in NCX forward mode displays the link between ranolazine-modulated cytosolic Na and Ca in such a way that both stimuli of the reverse NCX mode in heart failure, elevated [Na]_i_ and prolonged action potential duration, become attenuated by exposure to ranolazine, leading to improved Ca elimination during diastole. The results of this in vitro study led us to initiate the Ranolazine in Diastolic Heart Failure (RALI-DHF) trial (NCT01163734) to investigate intravenous ranolazine application in vivo followed by 2 weeks oral treatment in patients with diastolic dysfunction due to severe HFpEF in a small proof-of-concept placebo-controlled study (Fig. 2) [33].

Interestingly, isolated papillary muscles from transgenic mice overexpressing Ca/calmodulin-dependent protein kinase II (CaMKII), which is known to be upregulated in heart failure [34] and to induce late I_Na_, had frequency-dependent diastolic dysfunction [35]. Addition of ranolazine markedly improved diastolic tension under basal conditions but to a greater extent under frequency-induced stress, suggesting that CaMKII may be activated by increased Ca levels (and hence Ca overload) thereby activating late I_Na_ (Fig. 2). Indeed, direct effects of CaMKII on Na channels (and thus late I_Na_) were described previously [36] and underlined by computational modeling [37] and even effects of reactive oxygen species on late I_Na_ were recently shown to be mediated by CaMKII [38].

Our findings are consistent with earlier studies in animals showing that ranolazine attenuates diastolic dysfunction in the hearts of rabbit and rat models during ischemia/reperfusion [39, 40], in the presence of ischemic metabolites [41] or reactive oxygen species [42], and in dogs with experimentally induced heart failure [22]. In summary, most of the experimental studies performing acute exposure to ranolazine in heart failure report on positive effects on diastolic performance. This is different to a long-term study in heart failure dogs where both improved diastolic parameters and ejection fraction were described [31].

## Late I_Na_ Inhibition for the Treatment of Arrhythmias

Late I_Na_ is expected to influence electrophysiological cell properties in addition to cytosolic Na handling. Thus, it is not surprising that ranolazine has been shown to have beneficial effects on arrhythmias in vitro and in vivo. In general, ranolazine exerts antiarrhythmic capacities very likely via inhibition of late I_Na_, but also peak I_Na_ and rapid delayed rectifier potassium current I_Kr_ under certain circumstances. While inhibition of late I_Na_ is the principal electrophysiological effect of ranolazine in ventricular myocardium, the inhibition of peak I_Na_ seems to be of great importance in atrial myocardium.

A pathophysiologically increased late I_Na_ by itself can alter cellular electrophysiology by two distinguished ways and thus increase the propensity for arrhythmias, i.e. i) elevation of late I_Na_ which prolongs cardiac action potentials, and ii) elevation of late I_Na_ which causes cellular Na-dependent Ca overload and thus the electrogenic transient inward current I_Ti_.

Early afterdepolarizations are more likely to occur during a prolonged action potential duration, which can be induced by enhancing late I_Na_. Moreover, transmural differences of late I_Na_, and hence action potential duration, might increase transmural dispersion of repolarization and QT interval, which underlies the development of torsade de pointes arrhythmias [20].

In contrast, Ca overload, which occurs when late I_Na_ is elevated [12], and leakiness of the cardiac ryanodine receptor are believed to participate as crucial events in the initiation and propagation of spontaneous sarcoplasmic reticulum Ca release events and/or proarrhythmogenic Ca waves [43]. The consequence may be elimination of cytosolic Ca via the NCX, which generates I_Ti_, which can give rise to delayed afterdepolarizations [44]. Song and coworkers [45] have shown the crucial importance of late I_Na_ for arrhythmias in guinea pig atrial myocytes. They observed early and delayed afterdepolarizations as well as triggered activity. Also, elevation of late I_Na_ induced a Ca-dependent I_Ti_. All effects could be abolished by ranolazine or tetrodotoxin. Moreover, Ca chelating agents, Na/Ca-exchange blockers, and the sarcoplasmic reticulum Ca release inhibitor ryanodine could prevent delayed afterdepolarizations and triggered activity. Because early afterdepolarizations could not be prevented using these agents, it is suggested that action potential prolongation causes early afterdepolarizations in a Ca-independent manner.

First results of antiarrhythmic properties of ranolazine were reported in a guinea pig in vitro model of long-QT syndrome 3 [46] and in the presence of ATX-II [47], a selective inducer for late I_Na_. In these studies, ranolazine reduced both afterdepolarizations as arrhythmic triggers and the transmural and temporal dispersion of repolarization as an arrhythmic substrate. Therefore, the efficacy of ranolazine was potentially ascribed to its late I_Na_-blocking properties. Further studies underlined the antiarrhythmic effects of ranolazine; intact rat hearts subjected to ischemia/reperfusion showed a reduced incidence and duration of ventricular arrhythmias upon ranolazine treatment [48]. Recently, ranolazine had efficacy against both pacing-induced re-entrant and multifocal ventricular fibrillation in isolated-perfused rat hearts with H_2_O_2_-mediated early afterdepolarizations and triggered activity [49•].

Moreover, ranolazine reversed abnormalities of repolarization (prolonged action potential duration, beat-to-beat variability, and dispersion of action potential duration and early afterdepolarizations) of ventricular myocytes from failing canine hearts [22]. Also, it was shown recently that atrial myocytes from mice with long QT 3 mutation with increased late I_Na_ show greatly increased action potential duration and early afterdepolarizations, and ranolazine reduced action potential duration [50••].

However, ranolazine also inhibits I_Kr_ in cardiac myocytes [20]. Blocking I_Kr_ causes prolongation of the ventricular action potential. Therefore, the net effect of ranolazine on the action potential is mainly driven by the relative magnitude of reductions in late I_Na_ (inward) and I_Kr_ (outward) currents during the repolarization period.

It should be noted that there are also reports showing antiarrhythmic effects of ranolazine under conditions without elevated late I_Na_. Antzelevitch et al. [20] revealed potent effects of ranolazine to suppress early afterdepolarizations in myocytes isolated from the middle of the left ventricular wall and Purkinje fiber preparations. Midmyocardial cells are known to have action potentials that prolong disproportionately relative compared to those of epi- or endocardial cell types in response to many QT-prolonging drugs [51, 52]. Moreover, these cells have the largest late I_Na_ while I_Kr_ is similar in all three cell types. Accordingly, ranolazine produces a preferential abbreviation of midmyocardial cell action potential duration, leading to a reduction in transmural dispersion of repolarization [20]. In contrast to other I_Kr_ blockers such as sotalol, extrasystolic activity and spontaneous torsade de pointes arrhythmias were never observed in this study. Their findings are in agreement with a report from an anesthetized dog model with chronic complete atrioventricular block in which ranolazine-attenuated torsade de pointes episodes induced by I_Kr_ blockers [53] and studies involving isolated guinea pig and rabbit hearts [46, 54].

## Late I_Na_ Inhibition for the Treatment of Atrial Fibrillation

Rhythm control remains important in the treatment of AF, but cannot be effectively achieved without the risk of potential side effects such as proarrhythmia, hypotension, or sometimes organ toxicity with current drugs (eg, dronedarone or amiodarone), except for ß-blockers. Thus, there is a demand for novel pharmacological strategies to treat AF. Ranolazine has potent effects on atrial arrhythmias (eg, AF) that are worth mentioning. In the MERLIN TIMI-36 trial a significant reduction of supraventricular tachycardias was observed in patients that were treated with ranolazine. Although a low incidence of AF was found, patients treated with ranolazine were less likely to have a new onset of AF. While 75 patients developed new AF in the placebo group, only 55 individuals had new-onset AF during treatment with ranolazine. However, this trial was not designed and statistically powered to investigate new onset of AF. Nevertheless, in addition to this remarkable finding during assessment of ranolazine’s safety, further studies are warranted.

Murdock et al. [55] investigated high-dose ranolazine as a pill in the pocket approach. They found that 72 % of the patients with paroxysmal AF converted to sinus rhythm after application of 2,000 mg of ranolazine (single dose). Although very promising, the limitation of this study is that no placebo collective was included. In another pilot project, ranolazine was helpful in maintaining sinus rhythm in patients with resistant AF in whom more established measures had failed [56].

Finally, there is preliminary evidence from a recent abstract that investigated the effects of ranolazine compared to amiodarone to prevent AF following bypass surgery [57]. In this retrospective trial, baseline characteristics such as age, important other diseases, drug pretreatment, and others were not statistical different between both groups. Ranolazine (generally 1,500 mg preoperatively followed by 1,000 mg twice daily for 10 days) was given to 111 patients and 145 patients were treated with amiodarone (generally 400 mg preoperatively followed by 200 mg twice daily for 10 days). Patients treated with ranolazine were significantly less likely to experience AF, with an incidence of 15 % compared to 26 % in patients treated with amiodarone. Although the result of this retrospective trial is promising, selection bias cannot be ruled out as a cause for the reduced incidence of AF with ranolazine. So far, clinical data seems to be promising but it is limited due to trial design and number of patients.

One major problem concerning Na channel blockers in the past is the fact that they can cause ventricular proarrhythmia as it was demonstrated in the Cardiac Arrhythmia Suppression Trial (CAST) trial [58]. Hence, atrial selective peak I_Na_ inhibition would be an attractive approach for the treatment of atrial rhythm disorders due to the lack of ventricular proarrhythmia. It has been reported that ranolazine acts as an atrial selective peak I_Na_ inhibitor [59] but it selectively inhibits late I_Na_ in ventricular myocytes [20]. While it remains unclear how ranolazine selectively inhibits late I_Na_ in ventricular myocytes, its capacity to inhibit peak I_Na_ in atrial myocytes was largely attributed to electrophysiological differences between atrial and ventricular myocardium [59]. In the latter report the authors have properly announced some atrial capacities that may account for the atrial selective profile (inhibition of peak I_Na_) of ranolazine which was formerly described as an inactivated state blocker [22]: i) the half inactivation voltage is 16 mV more negative in atrial compared to ventricular myocytes; and ii) a more depolarized resting membrane potential in atrial cells that is generally accepted.

Although ranolazine was described to act as an inactivated state blocker, recent studies suggest that it preferentially binds rather to open versus inactivated Na channels, staying trapped in the channel during inactivation and unbinding during resting state [23, 60]. During rapid recovery from inhibition at the resting state, inhibition of peak I_Na_ might be atrial selective independent of open or inactivated blocking properties of the agent. This might be explained by a smaller fraction of rested Na channels at resting membrane potential in atrial compared to ventricular myocytes.

Accordingly, we also have shown that ranolazine inhibits peak I_Na_ in human atrial myocytes [11••]. We further found that inhibition of Na channels by ranolazine was frequency dependent, such as the higher the frequency the higher the inhibition rate. Faster activation rates are associated with abolished diastolic intervals, and the slow repolarization of the action potential phase 3 causes a slower unbinding of ranolazine from the channel. This phenomenon causes accumulation of block at fast, but not at slow frequencies. This might be explained by the capacity of ranolazine to dissociate rapidly from the resting state of the Na channel and may be of therapeutic importance during high atrial frequencies as it is known for AF.

Because Burashnikov et al. investigated myocytes without electrical remodeling (but this usually occurs during AF), these data are limited to conditions where electrical remodeling has not taken place yet [59]. Therefore, we additionally investigated atrial myocytes from patients with chronic AF and showed significantly reduced peak I_Na_ density (~16 %) in AF versus sinus rhythm, which was accompanied by a 26 % lower expression of Na_v_1.5 while neuronal Na channel isoforms were upregulated [11••]. In contrast, late I_Na_ was significantly increased in myocytes from AF atria by about 26 %. In a second step we exposed myocytes to ranolazine and found a marked reduction of late I_Na_ by about 60 % in myocytes from patients with AF but only by about 18 % in myocytes from patients with sinus rhythm. Although late I_Na_ integral per beat decreases with increasing frequencies, the high frequency (and thus an increasing late I_Na_ integral per minute) during AF might largely counteract this effect, leading to Na-dependent Ca overload Thus, it is likely that these effects of ranolazine independent of the action potential duration are important in human AF where Ca overload is also present [61].

But what may be the net effect of ranolazine on action potential duration in patients with AF with shortened action potential duration (as usually shown in AF)? Although there is competition between effects of ranolazine on late I_Na_ and I_Kr_, which also determines action potential duration, we have observed that ranolazine rather prolongs action potential in human atrial myocytes (Maier L, unpublished). Furthermore, it was shown that ranolazine does not shorten but rather prolongs action potential duration in dog atrial myocytes [59]. This may have been due to the fact that ranolazine also inhibits I_Kr_. Inhibition of I_Kr_ would counteract this phenomenon leading to prolonged atrial refractoriness. Indeed, it was previously shown that ranolazine prolongs atrial action potential duration, which leads to elimination of diastolic intervals and a more depolarized takeoff potential at rapid rates [59]. This effect may further potentiate the atrial selectivity for peak I_Na_ inhibition and possibly the clinical effectiveness of ranolazine. Taken together, inhibition of late I_Na_ via ranolazine may possess beneficial effects on cellular Na-dependent Ca overload similar to its property to block peak I_Na_ and I_Kr_, which are accepted strategies for the treatment of AF.

Moreover, Kumar et al. [62] investigated atrial electrical properties of ranolazine in an intact porcine heart. They found that ranolazine increased atrial effective refractory period and prolonged conduction time in a frequency-dependent manner. These effects were more pronounced in the atria than in ventricle, as was also observed previously [59]. Intravenous application of ranolazine also decreased acetylcholine-induced AF duration, the dominant frequency of the arrhythmia, and tended to suppress reinduction of AF in a porcine model [63]. The same group further provided evidence that intrapericardial ranolazine exhibits striking atrial antiarrhythmic actions in the intact porcine heart [64]. This was evidenced by increases in refractoriness and here in AF inducibility.

Recently, Sicouri et al. [65] investigated possible synergistic effects of ranolazine in combination with chronic amiodarone on AF. Their data indicate that the combination of both agents produced an atrial-selective inhibition of Na channel parameters and prevented induction of acetylcholine-induced AF, that was much greater than treatment alone, and greater than the algebraic sum of the individual treatments. Therefore, this study points to a synergism of the effects of the two therapies. Regarding severe organ toxicity, which is regular produced by amiodarone, it is of special interest that dronedarone in combination with low doses of ranolazine similarly resulted in atrial-selective depression of sodium channel–dependent parameters and effective suppression of AF [66••].

It also has been shown that ranolazine exerts antiarrhythmic action in canine pulmonary vein sleeve preparations, where this agent caused a frequency-dependent inhibition of Na channel activity leading to post-repolarization refractoriness, conduction slowing, and suppression of triggered activity [67].

Finally, we do not know which AF patient might benefit most from ranolazine, and thus, there is a huge need for clinical studies to investigate the effects of ranolazine on persistent and paroxysmal AF. Therefore, two placebo-controlled studies were recently initiated, including the Ranolazine in Atrial Fibrillation Following An Electrical Cardioversion (RAFFAELLO) trial investigating ranolazine in patients with persistent AF after electric cardioversion and how efficient sinus rhythm can be maintained over a period of 4 months (www.ClinicalTrials.gov; NCT01534962). The HARMONY trial investigates a combination of ranolazine and low-dose dronedarone in patients with paroxysmal AF assessing AF burden (www.ClinicalTrials.gov: NCT01522651) [68].

## Conclusions

In summary, there are increasingly experimental and preclinical data for a beneficial role of ranolazine in diastolic dysfunction and cardiac arrhythmias in addition to its current antianginal role. We believe that further experimental studies and future clinical trials will shed light onto the potential impact and future indications for ranolazine to treat patients possibly with certain arrhythmias, forms of heart failure most likely with diastolic dysfunction and HFpEF.
